# Pembrolizumab plus lenvatinib in advanced endometrial cancer: case report and systematic review of lung toxicity

**DOI:** 10.3389/fonc.2023.1145986

**Published:** 2023-07-10

**Authors:** Nicoletta Staropoli, Angela Salvino, Federica Falcone, Valentina Farenza, Martina Costa, Giacomo Rossini, Francesco Manti, Antonella Crispino, Caterina Riillo, Domenico Ciliberto, Mariamena Arbitrio, Pierfrancesco Tassone, Pierosandro Tagliaferri

**Affiliations:** ^1^ Medical and Translational Oncology Unit, AOU Renato Dulbecco, Catanzaro, Italy; ^2^ Department of Experimental and Clinical Medicine, Magna Græcia University, Catanzaro, Italy; ^3^ Radiology Unit, AOU Renato Dulbecco, Catanzaro, Italy; ^4^ Institute for Biomedical Research and Innovation (IRIB), National Research Council of Italy (CNR), Catanzaro, Italy

**Keywords:** advanced endometrial cancer, immunotherapy, targeted therapy, pembrolizumab, lenvatinib, lung toxicity

## Abstract

**Background:**

The optimal strategy for the treatment of recurrent and/or advanced endometrial cancer is still undefined. Recently, despite the lack of any predictive biomarker, the combination of pembrolizumab with lenvatinib has improved survival outcomes. We here report the long-term management of lung toxicity in a patient with endometrial cancer, and we critically review the current therapeutic options for this disease.

**Results:**

A patient with heavily pretreated endometrial cancer took pembrolizumab plus lenvatinib for 1 year, achieving a persistent partial response with a time to treatment failure of 18 months, despite relevant lung toxicity that did not affect the remarkable overall clinical benefit. A systematic review of this combination underlines the efficacy outcome despite toxicity. Interestingly, the literature review on lung toxicity suggested the role of anti-angiogenetic agents in the pathogenesis of lung cavitation, probably related to direct treatment activity, and disclosed a potential radiological sign predictive of the activity of anti-angiogenetic agents.

**Conclusion:**

We underline the efficacy of pembrolizumab plus lenvatinib in the current treatment landscape of endometrial cancer, underscoring the relevance of a correct management of toxicity.

## Introduction

Endometrial cancer (EC) is the sixth most common malignancy in women in the world (1).

At present, two major pathological conditions have been recognized: endometrioid carcinoma (type I), which represents 80% of all cases, and non-endometrioid carcinoma (type II), which includes the remaining 10%–20%, including serous carcinoma, clear cell carcinoma, and carcinosarcoma (2).

Recently, The Cancer Genome Atlas (TCGA) project analyzed about 400 different EC genomes using array- and sequencing-based technologies, which allowed the re-classification of EC into four different clusters according to genomics: POLE ultra-mutated, MSI hyper-mutated, copy number low, and copy number high (serous-like). This new classification is characterized by high biological heterogeneity able to impact the efficacy of adjuvant treatments, especially for women with aggressive and/or advanced-stage tumors, or predicting different outcomes (e.g., the POLE ultra-mutated type has the best prognosis whereas copy number high has the worst survival outcome) (3, 4).

Germline mutation or hereditary impairment of MMR genes, including MLH1, MSH2, MSH6, PMS2, and EPCAM, determined at least 5%–10% of Lynch syndrome, while epigenetic alterations, such as hypermethylation of the MLH1 promoter, epigenetic inactivation of MSH2, or downregulation of MMR genes by microRNAs, cause inhibition of transcription and interference with the expression of MMR genes and DNA repair function (5–7). Although several pathways are involved in the pathogenesis and proliferation of this tumor, particularly PI3K/AKT/mTOR alterations and mismatch repair pathway (8, 9), there is no gold standard second-line recurrent and/or advanced EC treatment after the failure of platinum-based regimens. Therefore, the current treatment of metastatic setting is still based on conventional treatment in which carboplatin plus paclitaxel is the standard (10, 11).

Moreover, hormone therapy could be considered for low-grade ER-positive cancer in adjuvant or front-line settings; targeted therapy, including trastuzumab, for tumors with HER2 overexpression; immunotherapy for dMMR/MSI-H patients; and antiangiogenetic therapy such as bevacizumab in monotherapy or in combination with carboplatin and paclitaxel, and lenvatinib with pembrolizumab for non-dMMR tumors/non-MSI high (11–19).

In Keynote-775, the lenvatinib plus pembrolizumab combination confirmed efficacy data from a previous study (Keynote-146), in terms of both PFS and OS irrespective of MRR status (20, 21).

Lenvatinib is a multitargeted tyrosine kinase inhibitor selectively acting on VEGFR 1–3, FGFR 1–4, PDGFRα, KIT, and RET, but, unlike other TKIs, it is also active in FGFR inhibition (22). Pembrolizumab is a monoclonal antibody targeting programmed death-1 (PD-1), an immune checkpoint receptor expressed on tumor-infiltrating T cells; it interacts with programmed death ligand 1 (PD-L1) and programmed death ligand 2 (PD-L2)—expressed both on several human and on some tumor cells—and causes the inactivation of T cells, preventing T cell-mediated tumor cytolysis (23). Pembrolizumab was evaluated in gynecological cancer that progressed after standard therapy in two different studies, phase Ib Keynote-028 and phase II Keynote-158, obtaining 13.5% ORR and 48% ORR, respectively, and encouraging survival improvement (24, 25).

Drug combination emerged as a therapeutic strategy for advanced/recurrent EC in the phase Ib/II trial Keynote-146/Study 111 where lenvatinib plus pembrolizumab was associated with a 38.0% ORR irrespective of MMR status in patients with advanced previously treated (two or less prior therapies) EC (20, 26). Recent results of the multicenter, open-label, randomized phase III Keynote-775 confirmed these preliminary data in 827 advanced, recurrent EC patients after one previous platinum-based chemotherapy (no exposure to vascular endothelial growth factors or anti PD-1 therapy). Patients were randomly assigned to receive both lenvatinib 20 mg orally once daily plus pembrolizumab flat dose of 200 mg IV every 3 weeks or chemotherapy of the physician’s choice (doxorubicin 60 mg/m^2^ IV every 3 weeks or paclitaxel 80 mg/m^2^ IV weekly). They were stratified according to MMR status (130 in the dMMR population and 697 in the pMMR population) and among pMMR patients. Primary endpoints were PFS and OS, while secondary endpoints included ORR according to RECIST version 1.1, safety and side effects and health-related quality of life. All endpoints were assessed in the pMMR population and among all patients. This trial showed a significant benefit in terms of mPFS and mOS in both pMMR EC [mPFS: 6.6 vs. 3.8 months, HR 0.60 (95% CI, 0.50–0.72) *p* < 0.001; mOS: 17.4 vs. 12.0 months, HR 0.68 (95% CI, 0.56–0.84) *p* < 0.001] and overall population [mPFS: 7.2 vs. 3.8 months, HR 0.56 (95% CI, 0.47–0.66) *p* < 0.001; mOS: 18.3 vs. 11.4 months, HR 0.62 (95% CI, 0.51–0.75) *p* < 0.001]. ORR was 30.3% for lenvatinib plus pembrolizumab and 15.1% for chemotherapy for the pMMR population and 31.9% and 14.7%, respectively, in the overall population (20, 21).

We here report the use of lenvatinib plus pembrolizumab in a heavily pre-treated EC patient, not expressing actionable mutations at liquid biopsy, and who underwent immunotherapy and antiangiogenetic treatment with a long-lasting major response. We focus on the management of the lung toxicity in this patient.

## Case presentation

On August 2018, a 61-year-old woman with EC was taken cared of by our team, after diagnostic surgery and diagnosis of moderately differentiated endometrioid adenocarcinoma, ER +, CD10 -, AML +, IIC stage. According to the stage of disease, she received, from September to November 2018, adjuvant therapy with AUC5 carboplatin and 175 mg/m^2^ q21 paclitaxel for four cycles followed by 40 mg/m^2^ weekly cisplatin for five cycles, concomitant with pelvic external radiotherapy and vaginal brachytherapy. After 13 months from diagnosis [platinum-free interval (PFI): 10 months], in September 2019, the patient reported the first lung relapse and took carboplatin plus pegylated liposomal doxorubicine (PLD), for a total of six cycles, without improvement. To offer a personalized treatment, we performed a molecular profile with an FDA-approved NGS blood-based liquid biopsy (Foundation One® Liquid CDX). This *in vitro* diagnostic test was used for analyzing 311 genes including blood tumor mutational burden (TMB), microsatellite instability high (MSI-H), and tumor fraction values. In particular, it was performed for profiling a complete exonic sequence of 35 explored genes, introns of 7 genes involved in rearrangements, and select exons of an additional 35 genes. Unfortunately, no actionable mutations were found in this patient (27–29).

In particular, the absence of known biomarkers determined a condition of “not-addicted” EC, focusing on several factors: no PTEN and mismatch repair alterations were detected, BRCAwt, and no PI3k mutations. Moreover, the Dako test showed a lack of PD-L1 expression. After 6 months, the patient showed lung and peritoneal disease progression (PS ECOG 0) and was highly motivated to try new treatment options.

Taking into account the available data on pembrolizumab plus lenvatinib in “addicted-EC” and “not-addicted-EC” patients (Keynote-146), in June 2020, following our request, we were able to administer, through the AIFA national fund allowance, 200 mg ev d1q21 pembrolizumab plus 20 mg/day orally daily lenvatinib to this specific cancer patient (14, 30, 31).

The interesting preliminary Keynote-146 results encouraged this decision for a significant improvement in terms of ORR (37.2% in all populations, regardless of PD-L1 expression) as a potentially good approach compared to the less effective standard chemotherapy (32, 33).

**Figure 1:**
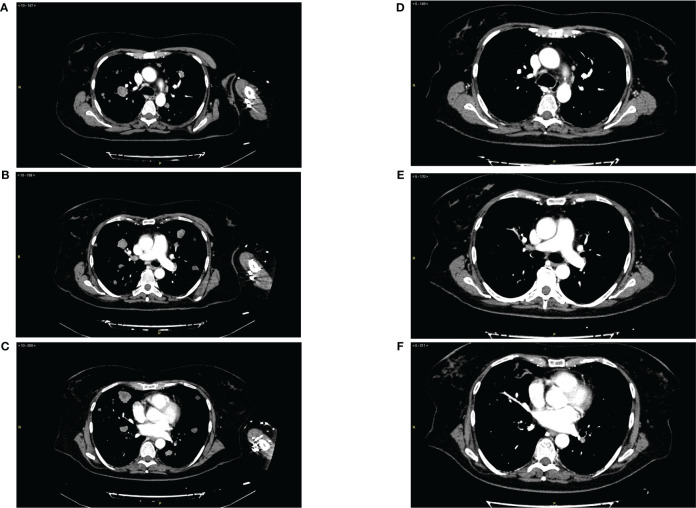
**(A–C)** Panel of radiological imaging concerning basal condition (June 2020) and presence of lung metastases. **(D–F)** Panel of radiological imaging concerning response to treatment (September 2020).

**Figure 2:**
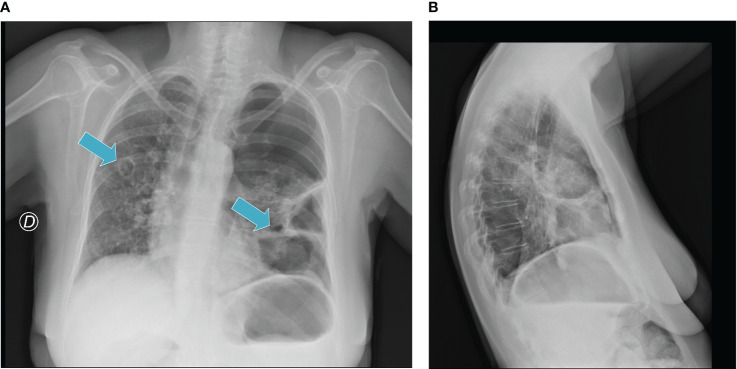
**(A, B)** Radiological evidence of “air-filled” cavitation.

The patient received this regimen for a total of 12 months (June 2020 to June 2021). Three months later (September 2020), a CT scan evaluation showed partial response with 80% reduction of lung metastases (**Figures 1A–F**) and appearance of some cavitations (**Figures 2A, B**) (34). At 6 months, CT scan showed a drastic change of lung imaging with evidence of cavitation in several lesions, previously described as a radiologic “air crescent sign” (35, 36). During treatment, we assessed routine blood test and tumor markers (CA 125, CA 15.3, and HE4) to follow tumor progression and treatment toxicity. At baseline, laboratory test and organ function results were as follows: hemoglobin 12.1 g/dl, platelet 311 x 10^3^/μl, neutrophils 7.0 x 10^3^/μl, and normal values of liver function and renal function. During treatment, no significant changes of these parameters were observed. At the time of progression, laboratory test results were as follows: hemoglobin 11.2 g/dl, platelet 570 x 10^3^/μl, neutrophils 23 x 10^3^/μl, and normal values of renal function and cholestasis index alterations (alkaline phosphatase 111 U/l and gamma glutamyl transferase 105 UI/l).

In particular, we focused on the trajectory of CA 125 and neutrophil-to-lymphocyte ratio (NLR) for a total evaluation of overall efficacy outcome and potential inflammation role. Indeed, NLR is a peculiar indicator of systemic inflammation and a potential parameter of immune response (37, 38).

**Figure 3:**
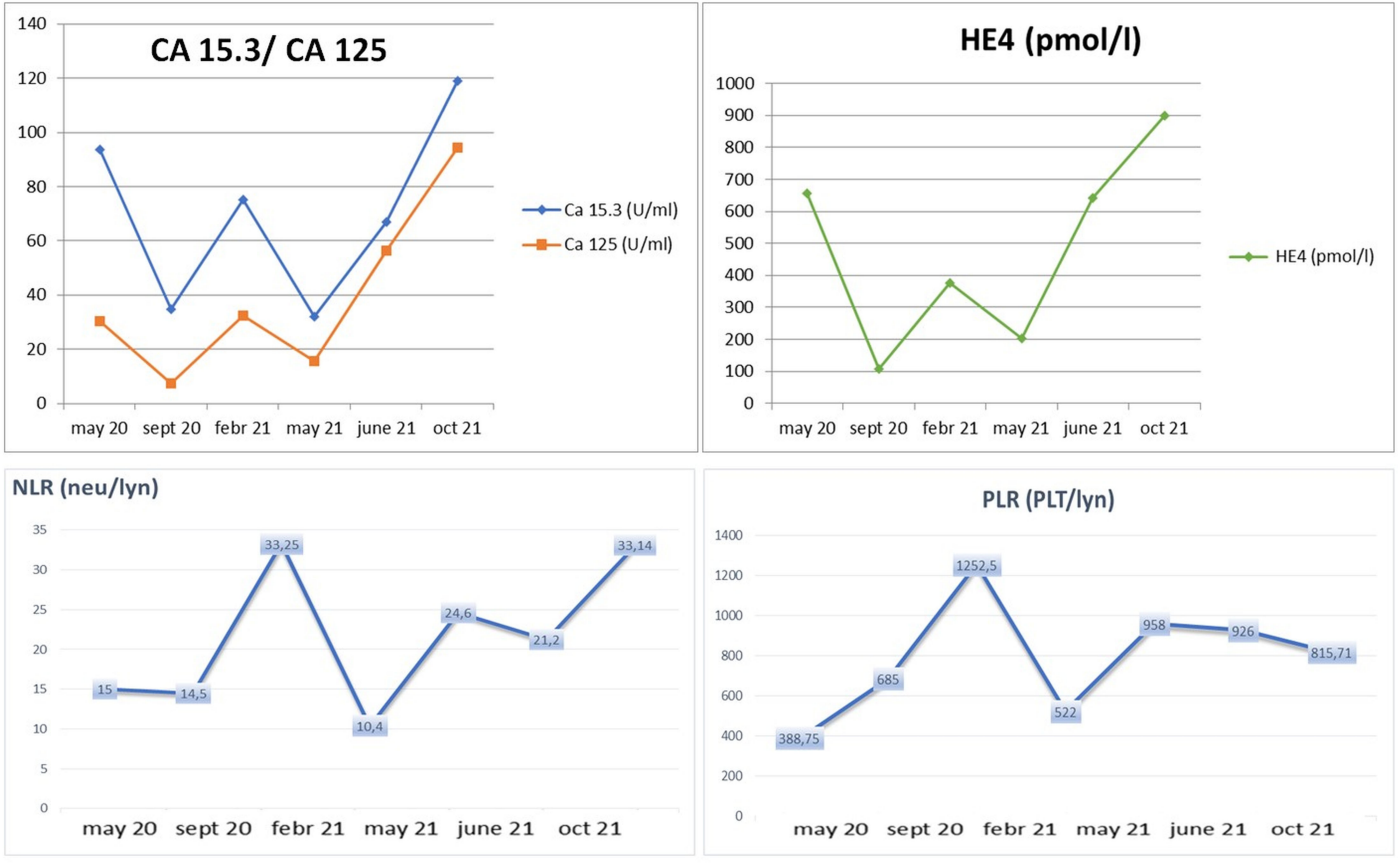
Clinical trend of biochemical markers (CA 125, CA 15.3, and HE4) and hematological inflammatory marker (NLR) according to response to treatment and “stop and go” strategy.

Interestingly, the CA 125/NLR curve disclosed an increase of these values after treatment discontinuation (**Figure 3**) (39).

In December 2020, the pulmonary toxicity with dyspnea and acute respiratory failure required hospital admission for left pneumothorax (grade IV CTCAE) with initial contralateral mediastinal slip and the patient underwent thoracic drainage. The treatment was temporarily withdrawn and then restarted in January 2021 with regression to grade I toxicity. However, we observed an overall tumor control for the entire treatment period and more: this particular outcome seems to relate to a potential immunotherapy “carryover effect”. In May 2021, despite clinical benefit, CT scan showed new cavitated lesions (the major lesion was 48 x 72 mm); thus, in order to avoid the risk of pneumothorax, the treatment was interrupted again in June 2021, maintaining partial response for almost 4 months. Finally, in October 2021, because of the lack of clinical improvement and owing to disease progression, based on high patient’s motivation, she underwent new treatment with weekly paclitaxel until disease progression and then the PS progressively declined until death.

**Figure 4:**
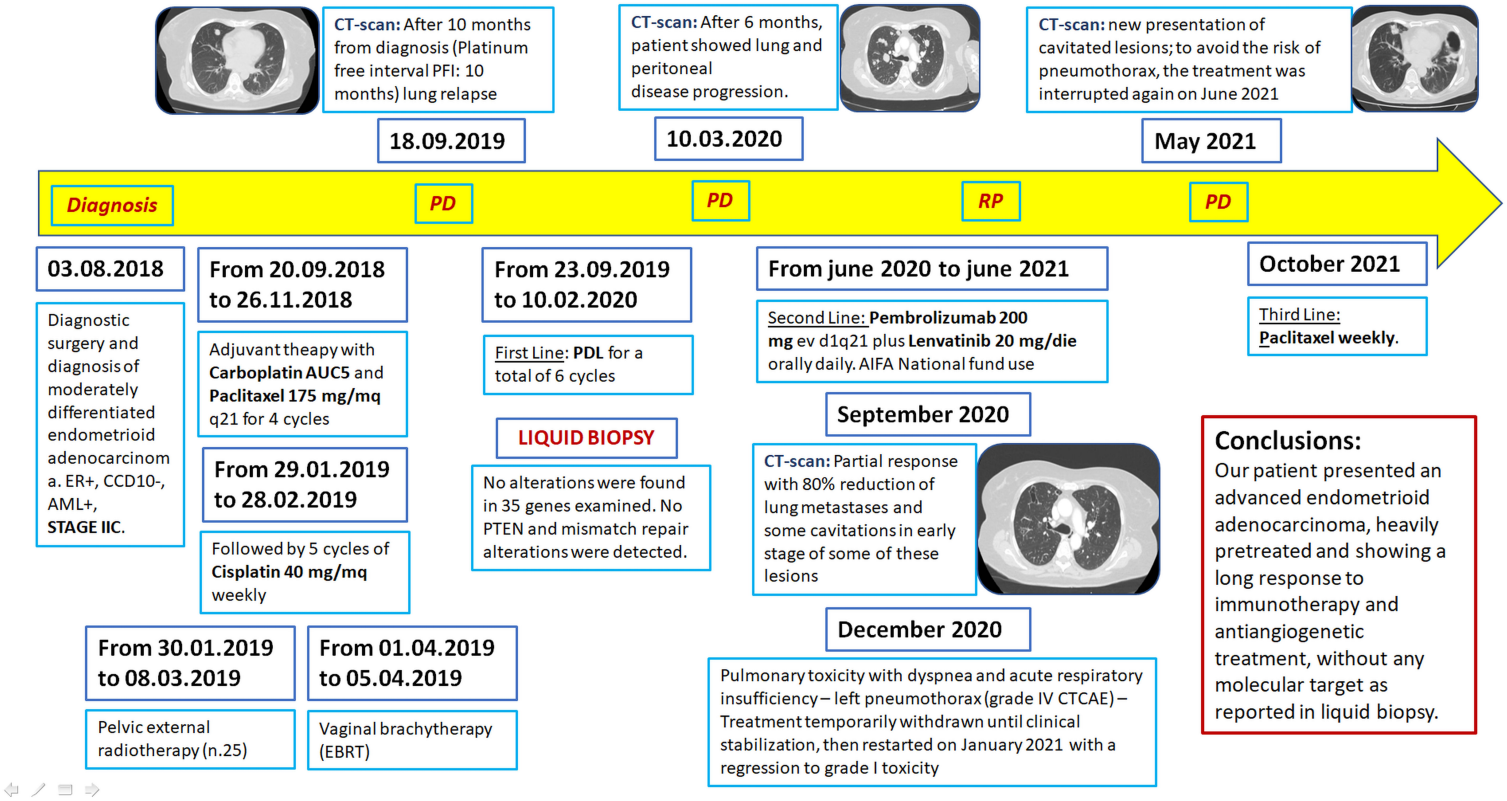
Treatment and history timeline.

Finally, **Figure 4** shows the timeline detailing case history by diagnosis to death with particular attention on clinical tests, lines of treatment, and disease progression.

### Systematic review on drug toxicity

After case report description, we focus on the management of lung toxicity in this patient.

## Methods

Case presentation was performed according to the CARE statement since the introduction of antiangiogenic agents in clinical practice, considering the improvement of the survival outcome in several solid tumors, the identification of new radiological response parameters has emerged (31). We performed a systematic review in order to select and describe all literature reports on this topic, according to the PRISMA statement.

### Searching

As previously performed, we conducted the bibliographic research on PubMed by using the search terms “anti-PD1”, “anti-PDL1”, “immunotherapy”, “anti-VEGF”, “antiangiogenetic therapy”, “lung toxicity”, and “lung cavitations”. The time frame was 2015–2022. For this search, only papers in English language were considered.

### Data extraction

The works were examined, independently, by investigators (M.C., G.R., and N.S.) to select homogeneous studies on lung toxicity reported as pembrolizumab toxicity, lenvatinib toxicity, or drug combination toxicity. The evaluation was extended to immunotherapy lung toxicity and antiangiogenetic drug-lung toxicity.

## Results

According to our selection, **Table 1** describes literature reports on lung toxicity (40–45). Several data described similar experience in terms of pulmonary toxicity related to the use of these drugs in different types of tumors. Murayama et al., evaluating 26 patients with anaplastic thyroid cancer with lung metastasis, underlined the prognostic value of lung cavitations in patients treated with lenvatinib [OS 186 days vs. 89 days for the cavitation (+) group and the cavitation (–) group, respectively] (41). Similarly, Kawanishi et al. reported a case of cavitation and pneumothorax in a 71-year-old man suffering from hepatocarcinoma (HCC) with lung metastasis and treated with lenvatinib (42). Although CT scan showed tumor size stability, they considered this event suggestive of a response to the treatment. Differently, Gennatas et al. showed how pneumothorax during TKI treatment (in this case with crizotinib for lung adenocarcinoma) can occur spontaneously, in the absence of cavitations of the lung metastasis (43). Furthermore, spontaneous pneumothorax was described during an anti-VEGF therapy with bevacizumab plus FOLFOXIRI for a metastatic colorectal cancer with lung metastases (45). Finally, gefinitib was associated with simultaneous bilateral spontaneous pneumothorax in a patient treated for lung adenocarcinoma with multiple parenchymal metastases (40).

**Table 1:** Systematic review of toxicity literature data according to PRISMA statement.

Trials (First Author)	Ref	Year	Design type	Tumor type	Population	N. of patients	Treatment	Toxicity	Follow up
Murayama D.	(32)	2022	retrospective study	anaplastic thyroid cancer	Asiatic	1	lenvatinib	lung cavitations	+
Kawanishi Y.	(33)	2020	case report	hepatocellular carcinoma	Asiatic	1	lenvatinib	pneumothorax	until death
Gennatas S.	(34)	2013	case report	lung adenocarcinoma	Caucasic	1	crizotinib	pneumothorax	–
Mori M.	(31)	2005	case report	lung adenocarcinoma	Asiatic	1	gefitinib	pneumothorax	+
Yang S.H.	(36)	2010	case report	colorectal adenocarcinoma	NS	1	bevacizumab and CHT	pneumothorax	–
Wang R.	(35)	2020	case report	lung adenocarcinoma	Asiatic	1	pembrolizumab	hemoptysis and cavitations	until death

Described Literature reports on lung toxicity (31–36).

Conversely, no instances of pulmonary toxicity (cavitation or pneumothorax) and immunotherapy, such as pembrolizumab, were reported in the literature. Pneumonitis is the only pulmonary disease of immune checkpoint inhibitors (frequency <5%). Moreover, an interesting report on unusual pulmonary toxicity was described in Beijing, where a patient suffering from non-small cell lung cancer (NSCLC) and treated with pembrolizumab presented an atypical lung toxicity with hemoptysis and cavitation, in the absence of other possible causes (44).

Consistent with literature description, our experience underlined the potential prognostic value of pulmonary toxicity for different types of solid tumors with or without lung metastasis, suggesting that toxicity may represent a predictor of long-term disease control. Cavitation of metastatic lung lesions, determining pneumothorax, can be the clinical presentation of this toxicity, but pneumothorax can even occur in patients with no lung metastatic lesions. Of the lung cancer patients who took bevacizumab the 14%–24% presented cavitation. This radiologic sign is described as a density modification of tumor mass reported as “air-filled”. This event, whose mechanism and prognostic role remain unclear, seems to correlate to response to therapy.

Conversely, the absence of comparative data regarding the disease control rate of patients with cavitated lesions, compared to patients who did not present cavitations, prevents us from identifying it as a predictive response marker. Indeed, the fragility of the patient, with a greater predisposition to infections, could result in a worse prognosis. A new point of view of our work is focused on the maintenance of efficacy despite a “stop and go” treatment administration. What we observed in this case is how pembrolizumab plus lenvatinib can be discontinued because of toxicity and, despite this discontinuation, how antitumor effect can be maintained. We had to stop the therapy twice in order to treat toxicity, but our patient continued to show clinical and radiological improvement.

## Discussion

The literature review showed several cases of lung toxicity related to systemic treatments, such as taxanes, tyrosine kinase inhibitors (TKIs), anti-VEGF agents, and immunotherapy, with a different clinical presentation: taxane-induced pneumonitis, TKI-associated interstitial lung disease, spontaneous pneumothorax by anti-VEGF drugs, and immunotherapy-related pneumonitis (46, 47).

In our experience, the metastatic EC patient treated with both immunotherapy and TKI in a second-line setting developed lung toxicity, characterized by cavitation and pneumothorax.

After 3 months of treatment, we observed cavitation of the metastatic lesions that caused left pneumothorax (shown in the chest radiograph in December 2020) with dyspnea and acute pulmonary failure. We treated the pneumothorax with thoracic drainage and the treatment was discontinued until clinical resolution (January 2021). The treatment was interrupted twice in May 2021 for a second episode of lung metastasis cavitation and in June 2021. In October 2021, treatment was withdrawn due to disease progression. Considering the important role of microenvironment regulation, several authors showed higher activity of anti-VEGF plus anti-PD-L1 combinatory approaches *in vitro* and *in vivo* (26). Based on this observation, we hypothesized the possible interaction between immunotherapy and antiangiogenetic therapy in this toxicity event. Antiangiogenetic therapy seems to reprogram the tumor immune microenvironment. Abnormal angiogenesis changes the function and expression of antitumor lymphocytes downregulating adhesion molecules, increasing interstitial fluid pressure and inducing hypoxia that upregulates some inhibitory signals for antitumor immune response (48). The ICI-ILD mechanism is still unclear. An increase of inflammatory cytokines could probably trigger the onset of irAEs. The inflammatory cytokine interleukin (IL)-6 induces the differentiation of naive CD4 T cells to Th17 cells, and it has been related to the incidence of irAEs (25). In particular, Th17 cells are involved in several autoimmune diseases (49, 50). Moreover, tumor necrosis factor-α (TNF-α) could be associated with irAEs. Conversely, a potential anti-angiogenic activity mediated by IFN-γ signaling that could condition the vascular endothelial cells’ function and promote T-cell infiltration was reported. Several works showed that lenvatinib reduced TAMs and increased CD8+ T cells (51). This condition could perhaps suggest negative feedback that is able to increase and regulate dendritic cell and macrophage function related to immunoinflammatory response.

## Conclusions

Our patient history represents an interesting example of a persistent response to immunotherapy and antiangiogenetic combination, beyond the toxicity interruption. To the best of our knowledge, it is the only case of “stop and go” treatment administration that configures a significant maintenance of efficacy, showing a durable response to treatment. Indeed, in this case, we observed how pembrolizumab plus lenvatinib can be discontinued because of toxicity, and despite this, its antitumoral effect can be maintained. We had to stop the therapy twice in order to treat and prevent toxicity, but our patient continued to show clinical and radiologic improvement. Conversely, according to the prognostic role of NLR, recognized as an inflammatory biomarker, the increase of this parameter (NLR 33 vs. NLR 15) was related to disease progression and dramatically occurred together with rapid clinical decline.

Our interesting result is consistent with literature data, if we consider that PD-L1 did not represent a predictive biomarker in EC as reported in the above-described clinical trials. Moreover, MMR status is an immunotherapy predictive biomarker in advanced EC, and the confirmatory Keynote-775 study supports the evidence of significant OS benefit in both pMMR-status patients and dMMR-status patients. Indeed, our patient showed long-term clinical benefit and a significant PFS advantage regardless of PD-L1 and MSI condition (20).

In future studies, the use of novel pharmacogenomics platforms may help in the identification of predictive biomarkers, which, in turn, will help in the understanding of biological events and in improving the quality of life and survival of these patients (52–55).

## Data availability statement

The datasets presented in this article are not readily available because Not available. Requests to access the datasets should be directed to not available.

## Ethics statement

All authors confirm that the patient provided and signed the informed consent that is authorized and approved in our Centre (Mater Domini Teaching Hospital), approved by our local ethical committee. Written informed consent was obtained from the participant for the publication of this case report.

## Author contributions

NS and PTg: conception and study design. NS, MC, GR, and FF: manuscript writing and sample collection. NS, DC, and MA: manuscript review and data analysis. AS and VF: manuscript review, teamwork had in charge of patients during treatment. PTg and PTs: supervision of manuscript review and in charge. All authors contributed to the article and approved the submitted version.
